# Breaching the Blood–Brain Interface: Vasoactive Neurons Contact Capillary Vessels of the Brain Clock in the Suprachiasmatic Nucleus

**DOI:** 10.1523/ENEURO.0401-25.2026

**Published:** 2026-04-30

**Authors:** Yifan Yao, Isabella Cannava, Ruya Tazebay, Saphira Samuels, Emily Silverstein, Rae Silver

**Affiliations:** ^1^ Department of Psychology, Columbia University, New York, New York 10027; ^2^ Department of Neuroscience and Behavior, Barnard College, New York, New York 10027; ^3^ Department of Pathology, and Cell Biology, Columbia University, New York, New York 10032; ^4^ Zuckerman Institute Affiliate, Columbia University, New York, New York 10027

**Keywords:** diffusible signal, glymphatic system, neurovascular connection, portal system, vasculature

## Abstract

The suprachiasmatic nucleus (SCN) produces diffusible signals sufficient to sustain circadian locomotor rhythms, although the nature of such signals, their targets, and the pathway whereby such signals may travel is unknown. It is possible that the venous portal veins that connect the capillary beds of the SCN to those of the organum vasculosum of the lamina terminalis (OVLT) provide a vascular pathway whereby signals originating in SCN neurons can reach local targets in the OVLT. Given the presence of the blood–brain interface (BBI) within the SCN, it is unclear how diffusible signals originating in SCN neurons might access the capillary vasculature of this nucleus. Estimates of astrocyte coverage of capillary vasculature range widely, from 70 to 100%, and furthermore such coverage can change dynamically. In the present study, we investigated whether three vasoactive peptidergic processes found in the mouse SCN, namely, vasopressin, vasoactive intestinal peptide, and gastrin-releasing peptide, might breach the BBI, thereby accessing capillary vessels. Using widefield and confocal imaging, we found neuron-to-capillary contacts between varicosities bearing each of these vasoactive peptides and capillary basal membranes, pericytes, and the endothelia in the mouse SCN of either sex. The findings suggest that all three vasoactive peptides may functionally breach the BBI of the SCN highlighting the importance of understanding how these peptides act on local vasculature to impact blood flow.

## Graphical abstract

**Visual Abstract:**
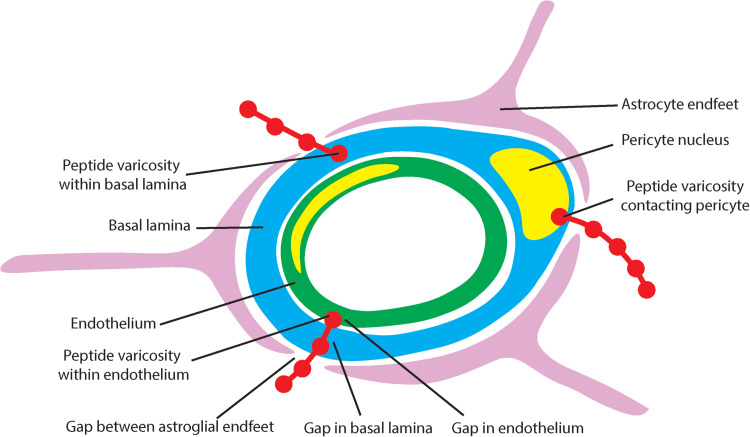
The capillary endothelium is surrounded by a basal lamina which has an inner and outer layer. Pericytes lie within the basal lamina between these two layers. Much but not all of the capillary basal lamina is surrounded by astrocytes. Gaps in astrocyte coverage of capillary vessels are loci where vasopressin-, gastrin releasing peptide-, and vasoactive intestinal polypeptide-positive processes access the basal lamina, pericytes and the capillary endothelium.

## Significance Statement

The suprachiasmatic nucleus (SCN) produces diffusible signals sufficient to sustain circadian locomotor rhythms. The SCN–organum vasculosum lamina terminalis portal pathway provides a route whereby signals of SCN origin might be relayed to the rest of the brain. The presence of the blood–brain interface (BBI), however, raises the question of how diffusible signals of SCN origin might access the capillary vasculature of the nucleus. High-resolution confocal imaging results suggest that varicosities of vasoactive peptides found in the SCN, namely, vasopressin, vasoactive intestinal peptide, and gastrin-releasing peptide, breach the BBI and directly contact capillary vessel compartments including basal laminae, pericytes, and endothelia.

## Introduction

The suprachiasmatic nucleus (SCN) serves as a brain clock, setting the phase of daily oscillation throughout the brain and body. The SCN signals the body via both neural efferents and extrasynaptic, diffusible signals (Ono et al., 2024). While SCN neural efferents have been amply studied, the pathway whereby diffusible neurosecretory signals reach their targets is unknown. Several lines of evidence indicate that diffusible signals are sufficient to sustain rhythms in circadian locomotor (Silver et al., 1996) and in extra-SCN electrical activity (Tousson and Meissl, 2004), but not in endocrine (Meyer-Bernstein et al., 1999).

One pathway whereby diffusible signals can reach their targets is by means of the vasculature, possibly via the portal blood vessels (BVs) linking the capillary beds of the SCN and the organum vasculosum of the lamina terminalis (OVLT; described in mouse, rat, and human; Yao et al., 2021; Roy et al., 2024; Yang et al., Under review). Importantly, in vivo studies show that the direction of blood flow is from the SCN to the OVLT (Roy et al., 2024). The joined capillary beds of the SCN and OVLT are positioned to enable signals of hypothalamic origin to reach local targets without dilution in the systemic blood supply, as occurs in the pituitary portal system (Popa and Fielding, 1930). The vascular organization of the SCN itself has been characterized in both rat and mouse (Ambach and Palkovits, 1975; Yao et al., 2023). The foregoing evidence of a portal vascular pathway from the SCN motivated the current effort to study the relationship between SCN neurons and their capillary blood vessels (BVs) in order to explore how diffusible output signals of the SCN might access local target sites via vascular routes.

Given the presence of the blood–brain barrier (BBB) in the SCN, the question arises as to how secretions of SCN neurons might access the capillary, thereby enabling diffusible signals to communicate time-of-day information to the OVLT. The BBB is a semipermeable membrane that is important in filtering substances exchanged between blood and the brain parenchyma (Wu et al., 2023). The anatomical components of the BBB include the BV endothelium, the tight and adherens junctions joining the endothelial cells, the basal lamina surrounding the endothelium, and the pericytes lying within the basal lamina. More recent understanding of the interface between brain and vasculature [i.e., blood–brain interface (BBI)] includes the concepts of the neurovascular unit, transporters, barrier cell secretions, adaptation, and modification of BBB functions (Banks, 2016). The best understood vasoactive peptides include the vasoconstrictor arginine vasopressin (AVP), and the vasodilators, vasoactive intestinal polypeptide (VIP) and gastrin-releasing peptide (GRP; Goadsby and MacDonald, 1985; Goldsmith, 1987; Clive et al., 2001), and these are abundant in the SCN (Moore et al., 2002). Each of these peptides has been implicated as an SCN diffusible output (Tousson and Meissl, 2004; Maywood et al., 2011). Furthermore, direct innervation of capillaries by AVP, GRP, and VIP in extra-SCN brain regions has been reported (Uddman et al., 1983; Jójárt et al., 1984; Paspalas and Papadopoulos, 1998). Neurons and their efferents bearing these peptides are spatially organized within the SCN. VIP neurons lie in the core region, AVP neurons lie in the shell, and GRP neurons are localized in a “cap” between the core and shell (Moore and Silver, 1998; Abrahamson and Moore, 2001; Moore et al., 2002). Fibers of these neurons are also spatially organized. AVP fibers lie predominantly in the shell. GRP fibers are densest in the cap and core regions. VIP fibers extend throughout the entire SCN.

Astrocytes and BVs are also spatially organized within the SCN. Glial fibrillary acidic protein-labeled astroglial processes in rat SCN are concentrated in the central area and weaker at rostral and caudal aspects, and some, but not all SCN neurons, are engulfed by astroglial processes (Tamada et al., 1998). With respect to BVs, whole BV length density, capillary length density, and surface area density are significantly greater in the shell than the core (Yao et al., 2023).

In the present study, we sought to determine whether AVP-, VIP-, and GRP-containing neurons innervate SCN capillaries. To this end, we surveyed the SCN using high-resolution confocal imaging of mouse brains labeled with AVP; VIP; GRP; two markers of the SCN vasculature, namely, basal lamina and endothelium; and DAPI for nuclei.

## Materials and Methods

### Animal and housing

C57BL/6NJ mice from The Jackson Laboratory aged 8–10 weeks were bred in the laboratory. All animals were provided with *ad libitum* access to food and water. The room was maintained at 21 ± 2°C and 35–70% humidity with a 12 h light/dark cycle (lights-on at 7:00 A.M.). All experiments were carried out in accordance with Columbia University's Institutional Animal Care and Use Committee protocol number AC-AABH1603.

### Perfusion protocol

Animals were deeply anesthetized (11% ketamine + 2% xylazine, 10 ml/kg body weight, i.p.) and perfused intracardially with 50 ml of 0.9% saline followed by 100 ml of 4% paraformaldehyde (PFA) in 0.1 M phosphate buffer at pH 7.3. Brains were postfixed at 4°C overnight and then cryoprotected in 20% sucrose until sectioning.

### Preparation of sections

For sectioning, the brains were embedded 6% and then 12% gelatin solution at 37°C for a minimum of 30 min. Once infiltrated with the gelatin, the brains were hardened at 4°C for at least 2 h. Next, the tissue was further hardened overnight in a solution of 4% PFA and 20% sucrose. Sagittal sections (50 µm) were cut on a cryostat (Leica Biosystems) and collected in 0.1 M phosphate-buffered saline (PBS). After washing in 0.1 M PBS 0.1% Triton X-100, sections were blocked with 1% normal donkey serum 0.1 M PBS 0.3% Triton X-100 for 1 h.

### Immunohistology

In order to identify SCN vasoactive neurons and their processes, antibodies to AVP, GRP, and VIP were used as follows: anti-AVP (rabbit, ImmunoStar, catalog #20069, lot #1922002), anti-VIP (rabbit, ImmunoStar, catalog #20077, lot #2006001), and anti-GRP (rabbit, ImmunoStar, catalog #20073, lot #1420001). In order to identify SCN BVs, Type IV Collagen-UNLB antibody (goat, SouthernBiotech, catalog #1340-01, lot #H2915-PI54) was used to label the basal lamina, and tomato lectin fluorescence (Vector Labs, catalog #FL-1171-1, lot #ZK0505) was used to label the endothelium. The following secondary antibodies were used: for AVP, VIP, and GRP, donkey anti-rabbit Cy3 (Jackson ImmunoResearch, code #711-165-152, lot #165051), and for collagen, donkey anti-goat Cy5 (Jackson ImmunoResearch, code #705-175-147, lot #160572). For each antibody, a dilution series was conducted to optimize the working concentration. The best signal-to-noise ratio was anti-AVP at 1:2,500, anti-VIP at 1:1,000, anti-GFP at 1:1,000, anti-collagen at 1:100, and lectin at 1:100. All primary antibodies were incubated with secondary antibodies at 1:200.

Antibody validation was performed using the following approaches. A primary-antibody-free control at each dilution tested was conducted to optimize the working concentration. No staining was observed in the absence of primary antibodies under any dilution. Antibody specificity was further assessed using preadsorption tests, by saturating antibodies with their corresponding antigens according to the manufacturer’s instructions. Tissues incubated with antigen-preabsorbed antibodies showed no staining. Finally, the staining patterns observed in the present study are identical to those reported in the published literature (Härtig et al., 2009; Lokshin et al., 2015; Varadarajan et al., 2018; Michalski et al., 2020; Taub et al., 2021).

### Microscopy and image processing

For widefield imaging, a Nikon Eclipse Ti2-E equipped with an Andor Zyla Plus sCMOS 4.2 MP USB-3 (water-cooled) and the following filters were used: Ex395/25 Dm425 Bar460/50 DAPI, Ex470/40 Dm495 Bar525/50 FITC/GFP, Ex545/25 Dm565 Em605/70 CY3, and Ex620/60 Dm660 Bar700/75 CY5. Capillaries were defined as BV segments of diameters smaller than 10 µm (Stefanovic et al., 2008) and neural varicosities as swollen elements along a fiber (∼0.5–2.5 µm; Ueda et al., 1983; Jacomy et al., 1999).

Next, the regions of interest were imaged with confocal microscopy. For confocal imaging, a SoRa-W1 Yokogawa spinning disk confocal microscope equipped with two Hamamatsu ORCA-Fusion sCMOS cameras and a 63× 1.46 NA Alpha Plan-Apochromat objective (Intelligent Imaging Innovations). Images were reconstructed in 3D with Imaris ver10.2 (Bitplane).

### Quantification

**Figure 1.**
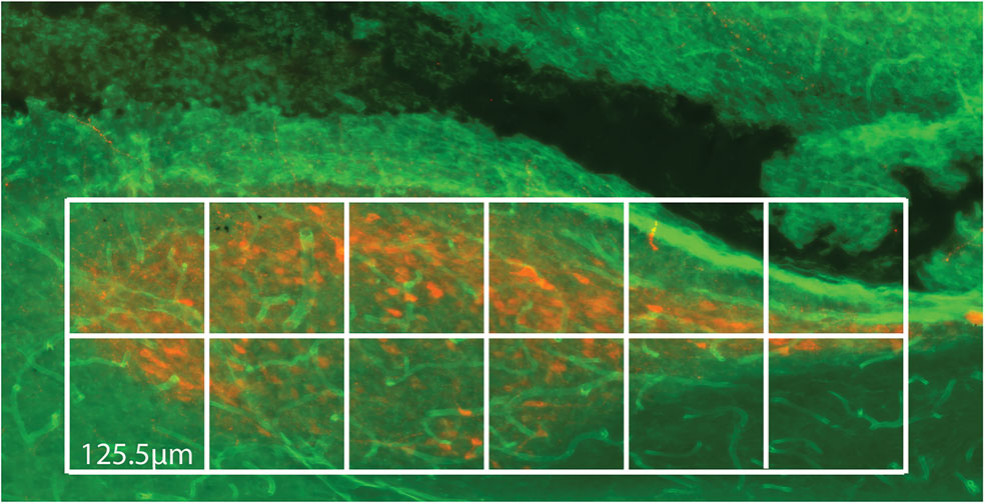
The SCN was compartmentalized into distinct units so as to divide the nucleus into squares of 125.5 × 125.5 µm^2^. Each square was examined for contacts of peptide varicosities with the capillary wall.

The following numbers of animals were studied for each peptide: AVP *N* = 6 (three females, three males), GRP *N* = 4 (three females, two males), and VIP *N* = 7 (two females, five males). SCN capillary vessels and AVP, VIP, and GRP peptidergic fibers are not uniformly distributed in the SCN. In order to assess the entire extent of the SCN, it was imaged in the sagittal plane using a 20× 0.75 NA CFI Plan Apo Lambda objective. *z* stacks were used to generate an extended depth of focus (EDF) image. To systematically survey the SCN, an image of the nucleus was overlaid with a grid of squares (125.5 × 125.5 µm^2^ squares, Fig. 1), and two independent investigators examined an EDF image of each square in the grid for potential points of contact between varicosities and capillary vessels. Each of these regions of interest was then imaged with confocal microscopy. The number of confocal stacks imaged was 62 for AVP, 39 for GRP, and 70 for VIP.

## Results

**Figure 2.**
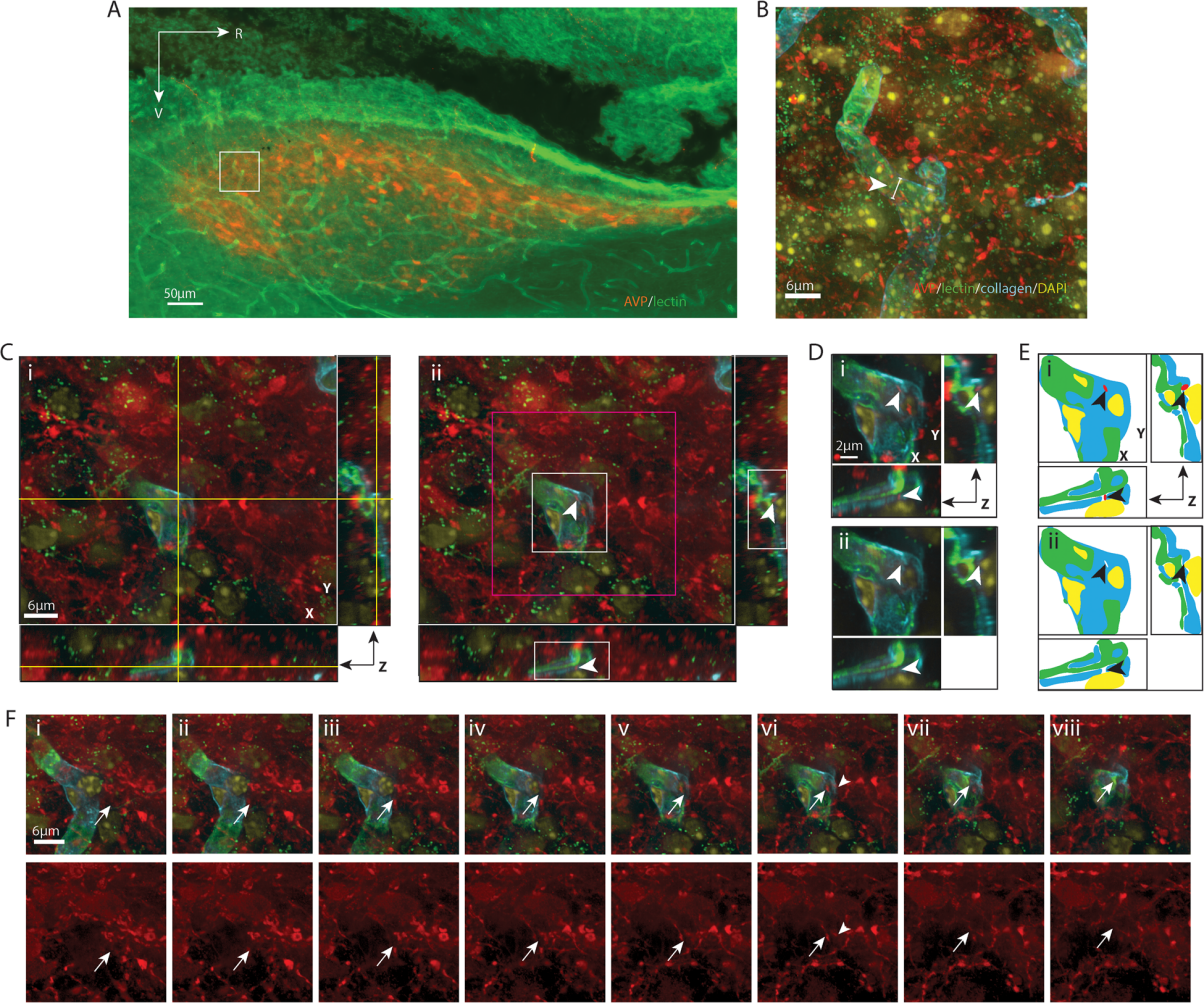
AVP varicosity lying in the capillary basal lamina matrix. ***A***, EDF image of the SCN in sagittal orientation. The boxed area is further examined using confocal microscopy in ***B–F***. The SCN is identified by AVP neurons, and the vascular endothelium is labeled with lectin. ***B***, Maximum intensity projection confocal image of the boxed area in ***A*** with additional labels for basal lamina (collagen) and nuclei (DAPI). AVP-positive fibers are seen coursing over a BV. The capillary segment (arrowhead), examined in the following panels, has a diameter of 5.05 µm. ***C***, An AVP-positive varicosity lying in the basal lamina is shown in all three orientations in an optical section. ***i***, The crosshair overlies the varicosity in the basal lamina. ***ii***, The crosshair is removed to enhance visualization of the varicosity (arrowhead), which has a diameter of 0.72 µm. Higher magnification images of the white and magenta boxed areas are further examined in ***D*** and ***F***, respectively. ***D***, To visualize the varicosity lying within the vascular wall, a magnified view of its localization is shown in three orientations. ***i***, AVP, collagen, lectin, and DAPI staining are merged to show that the varicosity (arrowheads) is in the basal lamina, residing at a curvature point of the capillary segment, and without penetrating into the endothelium layer. ***ii***, Removal of AVP reveals a gap (arrowheads) in the collagen staining where the varicosity resides. ***E***, Cartoon provides an illustration of key elements in ***D***. ***F***, Serial optical sections show the pathway of an AVP fiber (arrows) above (***i*–*v***) and below (***vii***, ***viii***) a varicosity (***vi***) described in the boxed area in ***C*** as it enters and exits the basal lamina. Top row, Four channels are merged to show the relationship between the fiber and BV wall. Bottom row, The AVP-only channel improves visualization of the fiber. ***i*–*iv***, A varicosity on the AVP-positive fiber appears. ***iii***, ***iv***, This fiber runs upward in the 11 o’clock direction. ***v***, ***vi***, The fiber branches and runs in the 2 o’clock direction. It penetrates the basal lamina. ***vii***, ***viii***, The fiber exits the BV wall. The arrowhead in ***vi*** serves as a location marker and points to the varicosity seen in ***C*** and ***D***. Color coding: AVP, red; lectin, green; collagen, blue; and DAPI, yellow. AVP, vasopressin; BV, blood vessel; EDF, extended depth of focus; SCN, suprachiasmatic nucleus. For ***B***, *z* = 15.1 µm; for ***C***, ***D***, and ***F***, *z* = 0.1 µm. ***F*** was taken every fourth section along the *z* stack.

**Figure 3.**
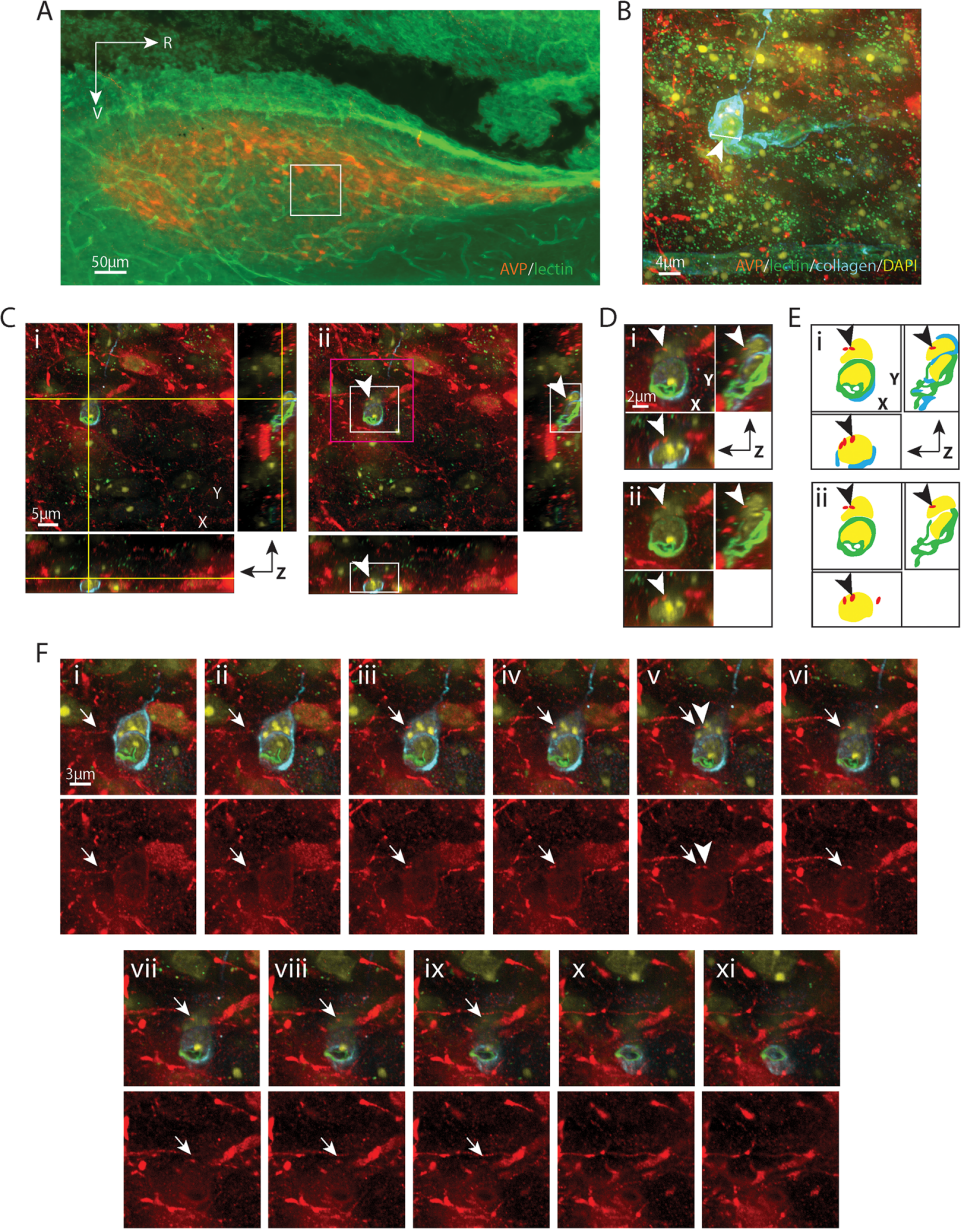
AVP varicosity contacting a pericyte nucleus. ***A***, EDF image of the SCN in sagittal orientation, labeled with AVP for SCN and lectin for endothelium. The boxed area is further examined with confocal microscopy in ***B–F***. ***B***, Maximum intensity projection of confocal image of the boxed area in ***A*** with additional labels of basal lamina (collagen) and nuclei (DAPI). Multiple AVP fibers are seen in close apposition to a capillary. The capillary segment (arrowhead), examined in the following panels, has a diameter of 5.79 µm. This segment is localized at the border between the core and the shell. ***C***, An AVP-positive varicosity, shown in all three orientations, contacts a DAPI-labeled nucleus in an optical section. ***i***, The crosshair denotes the location of a varicosity contacting the DAPI-labeled cell nucleus in the basal lamina. ***ii***, Removal of the crosshair enables better visualization of the varicosity (arrowhead), which has a diameter of 0.61 µm. Higher magnification images of the white and magenta boxed areas are further examined in ***D*** and ***F***, respectively, in order to follow the path of the fiber. ***D***, A magnified view in three orientations of a varicosity contacting the cell nucleus enables better visualization of the varicosity at the point where it contacts the BV wall. ***i***, AVP, collagen, lectin, and DAPI staining are merged to show that the varicosity is in the basal lamina. ***ii***, Removal of collagen shows that the top part of the DAPI-labeled nucleus contacts the AVP-positive varicosity and that its ventral aspect is covered by lectin labelled endothelium. Arrowheads point to the location of the varicosity as in ***C***. ***E***, Cartoon provides traces of key elements in ***D***. ***F***, Serial sections indicate the pathway of an AVP fiber (arrows) coursing above (***i*–*iv***) and below (***vi*–*xi***) a varicosity (***v***) that terminates in the basal lamina of the BV wall. Top row, Four channels are merged to show the relationship between the fiber and the cell in the basal lamina. Bottom row, The AVP-only channel allows better visualization of the pathway of the fiber. ***i*–*iv***, An AVP fiber reaches the capillary and enters the basal lamina. ***v***, The terminal of the AVP fiber contacts a DAPI-labeled nucleus surrounded by collagen. ***vi*–*ix***, the AVP-positive varicosity and the DAPI-stained nucleus gradually disappear together (***x***, ***xi***). The arrowhead in *v* serves as a location marker and points to the varicosity seen in ***C*** and ***D***. For ***B***, *z* = 16.02 µm; for ***C***, ***D***, and ***F***, *z* = 0.06 µm; ***F*** was taken every fourth section across the *z* stack. Remaining color coding and abbreviations as in Figure 1.

**Figure 4.**
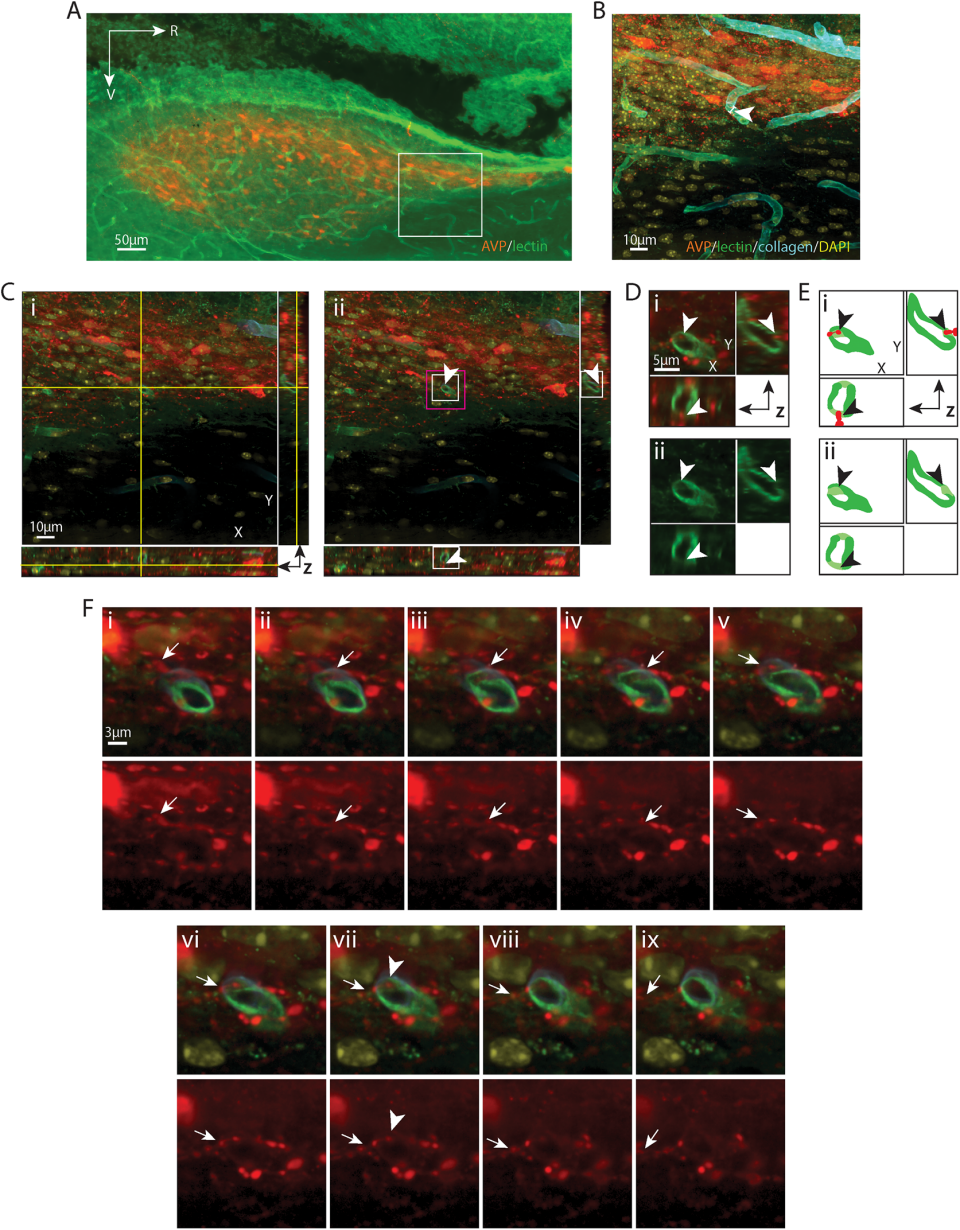
AVP varicosity lying in the capillary endothelium. ***A***, EDF image of the SCN in sagittal orientation, labeled with AVP for SCN and lectin for endothelium. The boxed area is further imaged with a confocal microscope in ***B–F***. ***B***, Maximum intensity projection of confocal image of the boxed area in ***A*** with additional labels of basal lamina (collagen) and nuclei (DAPI). The fibers and cell bodies of AVP neurons are seen entangled with capillaries. The capillary segment (arrowhead) examined in the following panels has a diameter of 6.59 µm. ***C***, An AVP-containing varicosity in the endothelium is shown in all three orientations (same area shown in B)****. ***i***, The crosshair denotes the location of the varicosity in the endothelium. ***ii***, Removal of the crosshair allows better visualization of the varicosity (arrowheads), which has a diameter of 1.13 µm. Higher magnification images of the white and magenta boxed areas are further examined in ***D*** and ***F***, respectively, in order to follow the path of the fiber. ***D***, Magnified view of the varicosity in all three orientations allows better visualization of the varicosity in relation to the vascular endothelium. ***i***, AVP and lectin staining are merged showing that the varicosity (arrowheads) is in the endothelium. ***ii***, The lectin-only channel shows a gap in lectin staining (arrowheads) where the varicosity resides, seen most clearly in the *xz* and *yz* planes. ***E***, Cartoon provides traces of key elements in ***D***. ***F***, Serial sections indicate the pathway of an AVP fiber (arrows) above (***i*–*vi***) and below (***viii*–*ix***) a varicosity (***v****ii*) which penetrates the endothelium. Top row, Merged images of AVP and BV showsthe pathway of the fiber. Bottom row, The AVP-only channel better displays the fiber. Arrows indicate the path of the fiber. ***i*–*iv***, A fiber crosses the collagen labeled basal lamina but does not yet access the endothelium at this point. ***v***, ***vi***, A varicosity of this AVP fiber lies close to the endothelium. ***vii***, The varicosity is seen in the endothelium. ***viii***, ***ix***, ***ix*–*xi***, The fiber exits the endothelium. The arrowhead in ***vii*** serves as a location marker and points to the varicosity seen in ***C*** and ***D***. Color coding and abbreviations as in Figure 1. For ***B***, *z* = 18.1 µm; for ***C***, ***D***, and ***F***, *z* = 0.1 µm; ***F*** was taken from every fourth section in the *z* stack.

**Figure 5.**
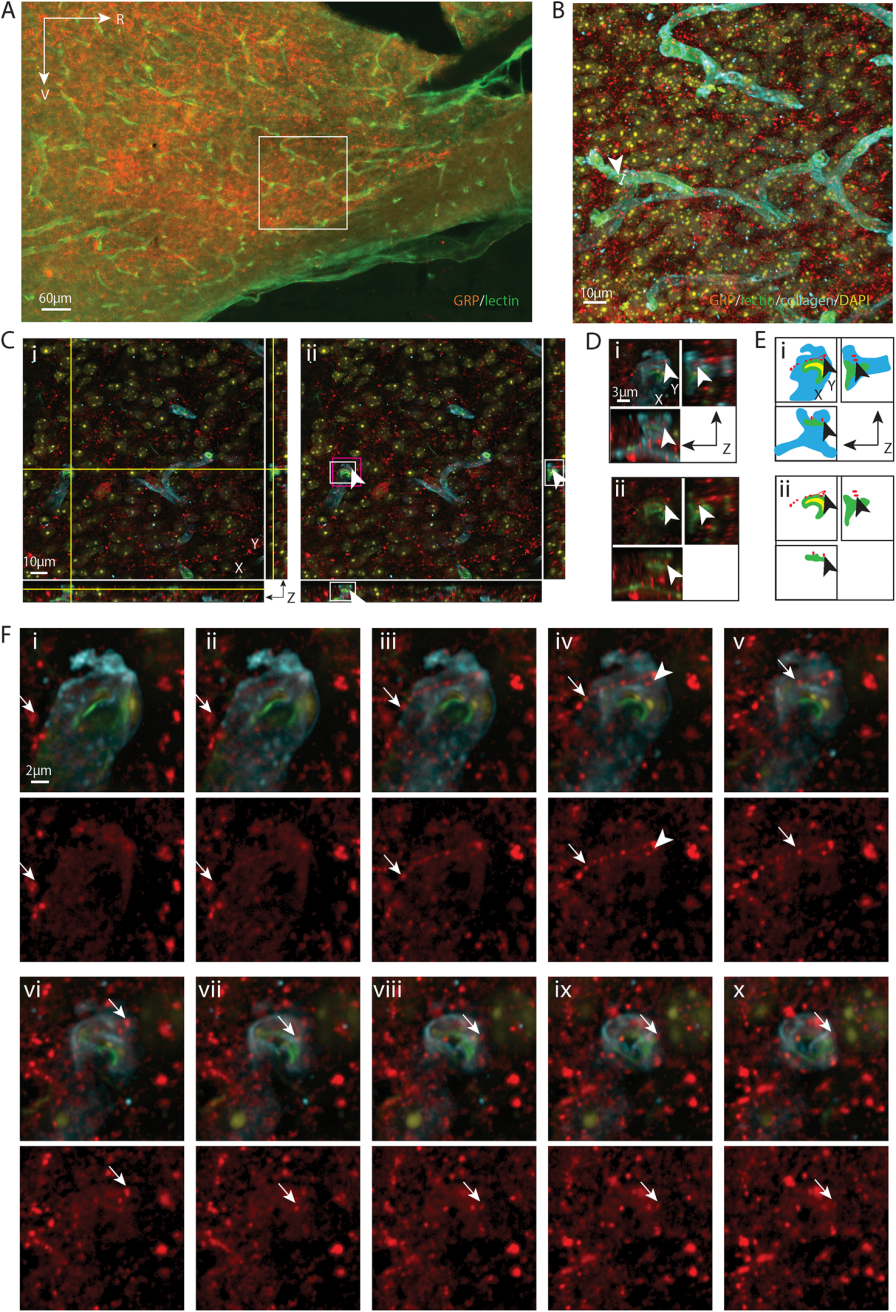
GRP varicosity lying in the capillary basal lamina matrix. ***A***, EDF image of the SCN in sagittal orientation, labeled with GRP for SCN and lectin for endothelium. The boxed area is further examined with confocal microscopy in ***B–F***. ***B***, Maximum intensity projection confocal image of the boxed area in ***A*** with additional labels of basal lamina (collagen) and nuclei (DAPI). Numerous GRP-positive fibers are seen coursing over the BV. The capillary segment (arrowhead) examined in the following panels has a diameter of 8.25 µm. ***C***, A GRP-positive varicosity lying in the basal lamina, shown in all three orientations, in an optical section taken from ***B***. ***i***, The crosshair denotes the varicosity in the basal lamina. ***ii***, Removal of the crosshair allows visualization of the varicosity (arrowhead), which has a diameter of 0.47 µm. Higher magnification images of the white and magenta boxed areas are further examined in ***D*** and ***F***, respectively, in order to follow the path of the fiber. ***D***, Magnified view in three orientations of the investigated varicosity to better visualize the varicosity in relation to the vascular wall. ***i***, GRP, collagen, lectin, and DAPI staining are merged to show that the varicosity is in the basal lamina. ***ii***, Removal of collagen shows the varicosity lying outside the territory of the endothelium. Arrowheads point to the location of the varicosity as in ***C***. ***E***, Cartoon provides traces of key elements in ***D***. ***F***, Serial optical sections show the pathway of a GRP fiber (arrows) above (***i*–*iii***) and below (***v*–*x***) a varicosity (***iv***) of the boxed area in ***C*** as it enters and exits the basal lamina, leaving five visible varicosities in the BV wall. Top row, Merged images of GRP and BV show the path of the fiber. Bottom row, The GRP-only channel enhances visualization of the fiber. ***i***, ***ii***, A GRP fiber segment with varicosities gradually appear and (***iii*–*v***) traverse the basal lamina horizontally. ***vi*–*ix***, The other end of the fiber exits the basal lamina and (***x***) gradually disappears. The arrowhead in ***iv*** serves as a location marker and points to the varicosity seen in ***C*** and ***D***. GRP (red), gastrin-releasing peptide; remaining color coding and abbreviations as in Figure 1. For ***B***, *z* = 17.1 µm; for ***C***, ***D***, and ***F***, *z* = 0.1 µm; ***F*** was taken from every fourth section in the *z* stack.

**Figure 6.**
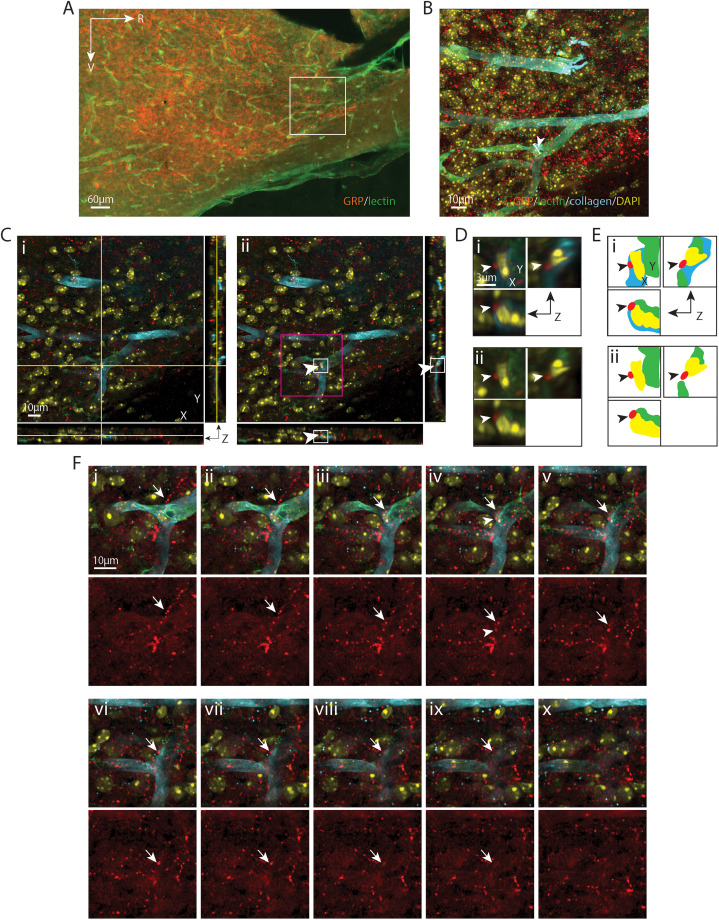
GRP varicosity contacting a pericyte nucleus. ***A***, EDF image of the SCN in sagittal orientation, labeled with GRP for SCN and lectin for endothelium. The boxed area is further imaged with a confocal microscope in ***B–F***. ***B***, Maximum intensity projection of confocal image of the boxed area in ***A*** with additional labels of basal lamina (collagen) and nuclei (DAPI). Numerous GRP fibers are seen coursing over the capillaries. The capillary segment (arrowhead) examined in the following panels has a diameter of 8.92 µm. ***C***, A GRP-positive varicosity, shown in all three orientations, contacts DAPI in an optical section taken from ***B***. ***i***, The crosshair denotes the location of varicosity contacting the DAPI-labeled cell nucleus in the basal lamina. ***ii***, The crosshair is removed to enhance visualization of the varicosity (arrowhead), which has a diameter of 0.72 µm. Higher magnification images of the white and magenta boxed areas are further examined in ***D*** and ***F***, respectively, in order to follow the path of the fiber. ***D***, Magnified view in three orientations of the varicosity contacting the cell nucleus to better visualize the varicosity in relation to the vascular wall. ***i***, GRP, collagen, lectin, and DAPI staining are merged to show that the varicosity is in the basal lamina. ***ii***, Removing collagen shows left half of the cell nucleus contacted by the GRP-positive varicosity is not covered by collagen and its right aspect is covered by lectin, indicating that the cell is localized within the basal lamina. The tip of the varicosity touches the nucleus on all three orientations, and its main body is localized in the basal lamina. Arrowheads point to the location of the varicosity as in ***C***. ***E***, Cartoon provides traces of key elements in ***D***. ***F***, Serial sections indicate the pathway of a GRP fiber (arrows) coursing above (***i*–*iii***) a varicosity (***iv***) that terminates on a cell in the basal lamina (***v*–*x***). Top row: Merged images of GRP and BV show the path of the fiber. Bottom row: The GRP-only channel enhances visualization of the fiber. ***i*–*iii***, A GRP fiber reaches the capillary from the 1 o’clock direction and enters the basal lamina. ***iv*–*vi***, The varicosity contacts a DAPI-labeled cell nucleus surrounded by collagen. ***vii*–*x***, The staining of the varicosity and the cell where the varicosity resides gradually disappear together. Arrows point to the fiber terminating on the basal lamina cell. The arrowhead in ***iv*** points to the same varicosity as in ***C*** and ***D***. Color coding and abbreviations as in Figure 4. For ***B***, *z* = 18.1 µm; for ***C***, ***D***, and ***F***, *z* = 0.1 µm; ***F*** was taken from every fourth section across the *z* stack.

**Figure 7.**
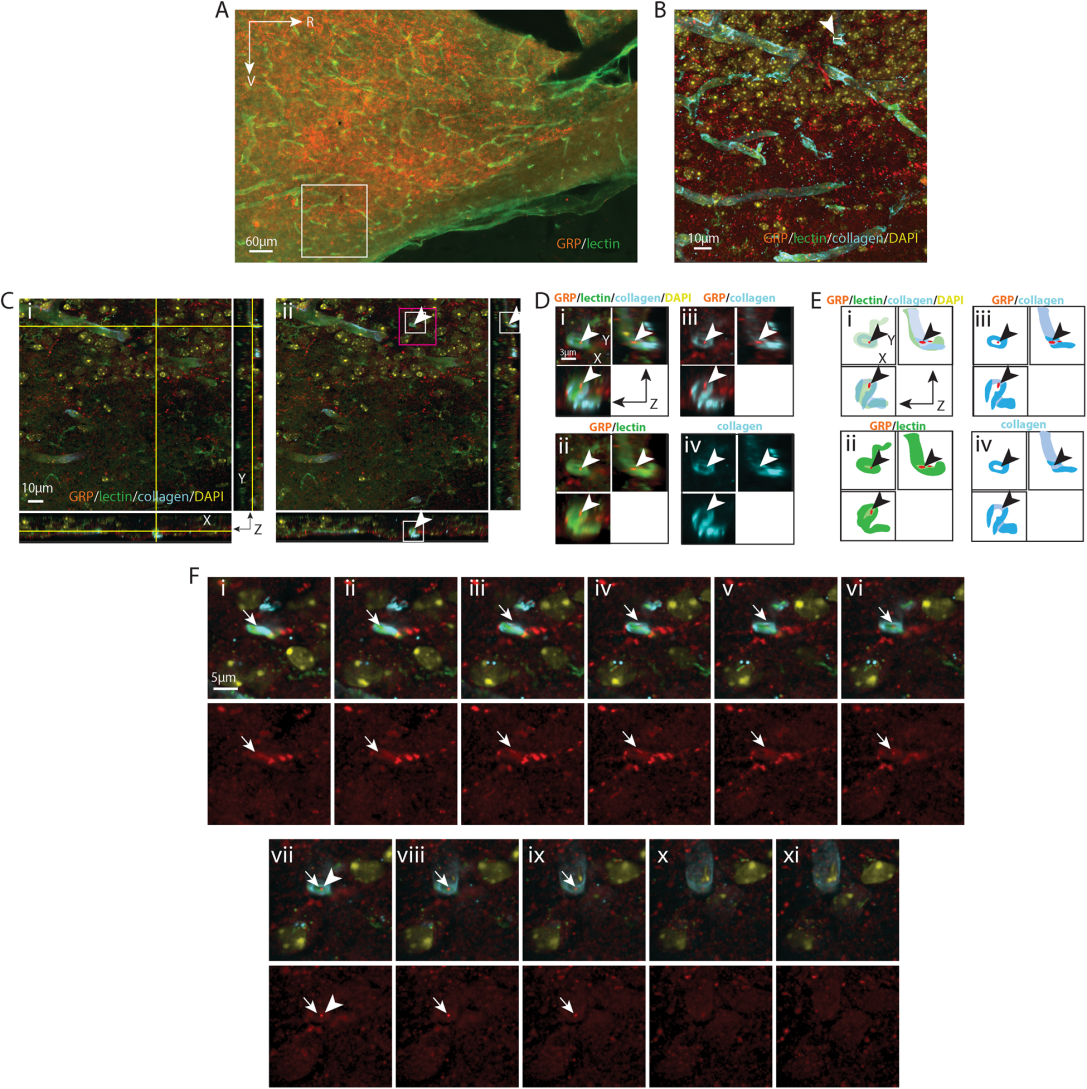
GRP varicosity lying in the capillary endothelium. ***A***, EDF image of the SCN in sagittal orientation, labeled with GRP for SCN and lectin for endothelium. The boxed area is further imaged with a confocal microscope in ***B–F***. ***B***, Maximum intensity projection of confocal image of the boxed area in ***A*** with additional labels of for basal lamina (collagen) and nuclei (DAPI). GRP fibers are seen intertwined with capillaries. The capillary segment (arrowhead) examined in the following panels has a diameter of 7.90 µm. ***C***, A GRP varicosity in the endothelium is shown in all three orientations in an optical section taken from ***B***. ***i***, The crosshair denotes the location of the varicosity in the endothelium. ***ii***, Removal of the crosshair allows better visualization of the varicosity (arrowheads), which has a diameter of 0.46 µm. Higher magnification images of the white and magenta boxed areas are further examined in ***D*** and ***F*** respectively, in order to follow the path of the fiber. ***D***, Magnified view in all three orientations allows visualization of the varicosity in relation to the vascular endothelium. ***i***, GRP, collagen, and lectin staining are merged to show the varicosity (arrowheads) in the endothelium. ***ii***, collagen and lectin channels are merged showing that a GRP varicosity lies in the gap of lectin staining (arrowheads). ***iii***, the GRP and collagen channels are merged showing that the GRP varicosity is not localized to the basal lamina as it lies in a “cavity” (arrowheads) lacking collagen staining. ***iv***, The collagen-only channel allows better visualize the cavity (arrowheads) in the basal lamina. ***E***, Cartoon provides traces of key elements in ***D***. ***F***, Serial sections indicate the pathway of a GRP fiber (arrows) above (***i*–*vi***) and below (***viii*–*xi***) a varicosity (***vii***) which terminates in the endothelium. Top row, Merged images of GRP and BV show the pathway of the fiber. Bottom row: The GRP-only channel allows better visualization of the fiber. ***i*–*v***, The varicosities of a GRP fiber segment gradually appear and surround a capillary vessel. ***vi*–*viii***, A varicosity on this fiber is seen in the endothelium. ***ix*–*xi***, The varicosity gradually disappears. The arrowhead in ***vii*** serves as a location marker and points to the varicosity seen in ***C***, ***D***. Color coding and abbreviations as in Figure 4. For ***B***, *z* = 21.1 µm; for ***C***, ***D***, and ***E***, *z* = 0.1 µm; ***F*** was taken from every fourth section across the *z* stack.

**Figure 8.**
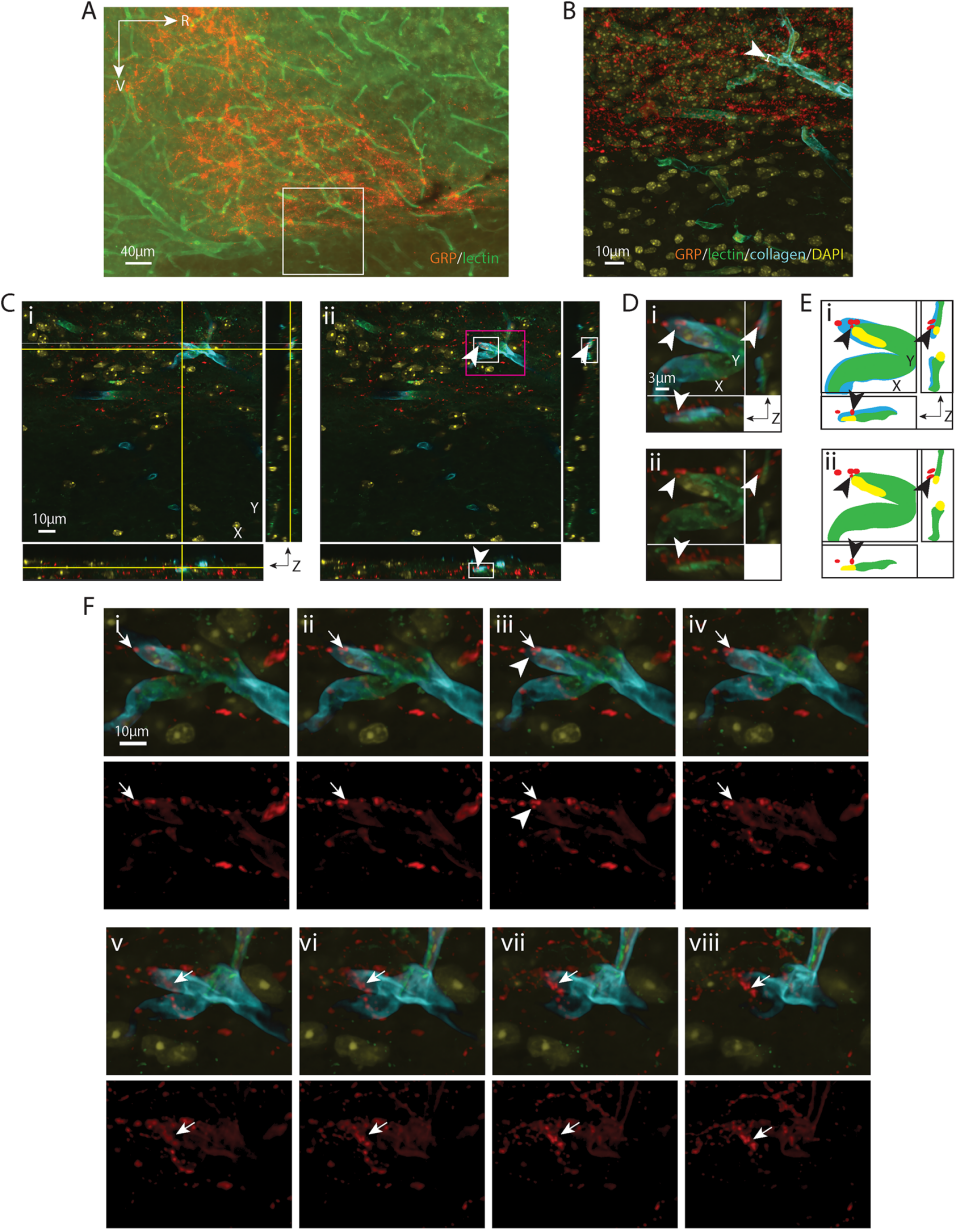
VIP varicosity lying in the capillary basal lamina matrix. ***A***, EDF image of the SCN in sagittal orientation, labeled with VIP for SCN and lectin for endothelium. The boxed area is further imaged with a confocal microscope in ***B–F***. ***B***, Maximum intensity projection of confocal image of the boxed area in ***A*** with additional labels of for basal lamina (collagen) and nuclei (DAPI). VIP-positive fibers are seen coursing over the BV. The capillary segment (arrowhead) examined in the following panels has a diameter of 5.57 µm. ***C***, A VIP-positive varicosity lying in the basal lamina, shown in all three orientations, in an optical section taken from ***B***. ***i***, The crosshair denotes the varicosity in the basal lamina. ***ii***, Removal of the crosshair enables better visualization of the varicosity (arrowhead), which has a diameter of 0.57 µm. Higher magnification images of the white and magenta boxed areas are further examined in ***D*** and ***F*** respectively, in order to follow the path of the fiber. ***D***, Magnified view in three orientations allows visualization of the varicosity in relation to the basal lamina and vascular wall. ***i***, VIP, collagen, lectin, and DAPI staining are merged to show that the varicosity is in the basal lamina. ***ii***, Removal of collagen shows the varicosity lying outside the territory of the endothelium. Arrowheads point to the location of the varicosity as in ***C***. ***E***, Cartoon provides traces of key elements in ***D***. ***F***, Serial optical sections show the pathway of a VIP fiber (arrows) above (***i***, ***ii***) and below (***iv*–*viii***) a varicosity (***iii***) of the boxed area in ***C*** as it enters and exits the basal lamina. Top row: Merged images of VIP and BV display the path of the fiber. Bottom row: The VIP-only channel allows better visualization of the fiber. ***i***, ***ii***, A VIP fiber reaches the BV. ***iii***, ***iv***, The fiber branches and the varicositie senter the BV basal lamina. ***v*–*viii***, The branch further extends and exits the BV wall. The arrowhead in ***iii*** serves as a location marker and points to the varicosity seen in ***C***, ***D***. Color coding and abbreviations: VIP, red; VIP, vasoactive intestinal peptide; the remaining are as in Figure 1. For ***B***, *z* = 23.1 µm; for ***C***, ***D***, and ***F***, *z* = 0.1 µm; ***F*** was taken from every fourth section across the *z* stack.

**Figure 9.**
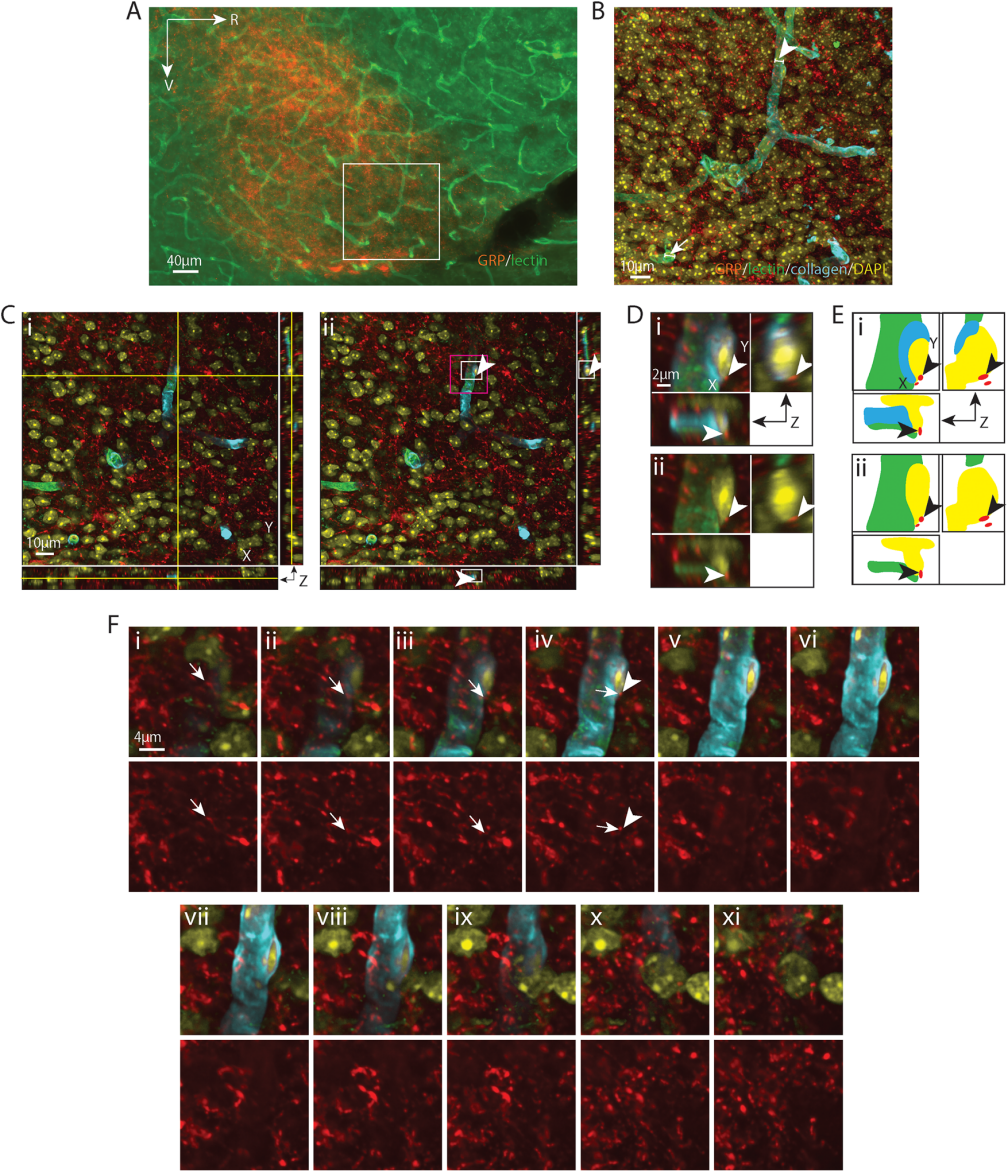
VIP varicosity contacting a pericyte nucleus. ***A***, EDF image of the SCN in sagittal orientation, labeled with VIP for SCN and lectin for endothelium. The boxed area is further imaged with a confocal microscope in ***B–F***. ***B***, Maximum intensity projection of confocal image of the boxed area in ***A*** with additional labels of basal lamina (collagen) and nuclei (DAPI). VIP fibers are seen coursing over the capillaries. The capillary segment examined in Figure 8 (arrowhead) has a diameter of 6.05 µm. The capillary segment examined Figure 9 (arrow) has a diameter of 4.39 µm. ***C***, A VIP-positive varicosity, shown in all three orientations, contacts DAPI in an optical section taken from ***B***. ***i***, The crosshair denotes the location of varicosity contacting the DAPI-labeled cell nucleus in the basal lamina. ***ii***, Removal of the crosshair enables better visualization of the varicosity (arrowhead), which has a diameter of 0.53 µm. The varicosity touches a cell nucleus localized in the capillary wall, protruding from the trunk. Higher magnification images of the white and magenta boxed areas are further examined in ***D*** and ***F***, respectively, in order to follow the path of the fiber. ***D***, Magnified view in three orientations allows visualization of the varicosity in the basal lamina. ***i***, VIP, collagen, lectin and DAPI staining are merged to show the varicosity contacting the nucleus. Half of the perimeter of the nucleus borders the endothelium in the *xy* orientation, but in the *xz* and *yz* orientations, the nucleus is separated from the endothelium by the basal lamina. ***ii***, Removal of collagen channel shows that the right half of the cell nucleus contacted by the VIP-positive varicosity is not covered by collagen and that its left aspect is covered by lectin. Arrowheads point to the location of the varicosity as in ***C***. ***E***, Cartoon provides traces of key elements in ***D***. ***F***, Serial sections indicate the pathway of a VIP fiber (arrows) coursing above (***i*–*iii***) a varicosity (***iv***) that terminates on a cell in the basal lamina (***vi*–*xi***). Top row, Merged images of VIP and BV show the path of the fiber. Bottom row, The VIP-only channel enhances visualization of the fiber. ***i*–*iii***, A VIP fiber appears and reaches the capillary and enters the basal lamina. ***iv***, The terminal of VIP fiber touches a DAPI-labeled cell surrounded by collagen whereas the fiber entry point is not covered by collagen. ***v*–*vii***, The varicosity disappears, and the cell is fully wrapped by the collagen. ***viii*–*xi***, The blood vessel gradually disappears. The arrowhead in ***iv*** serves as a location marker and points to the varicosity seen in ***C***, ***D***. Color coding and abbreviations as in Figure 7. For ***B***, *z* = 14.1 µm; for ***C***, ***D***, and ***E***, *z* = 0.15 µm; ***D*** was taken from every fourth section across the *z* stack.

**Figure 10.**
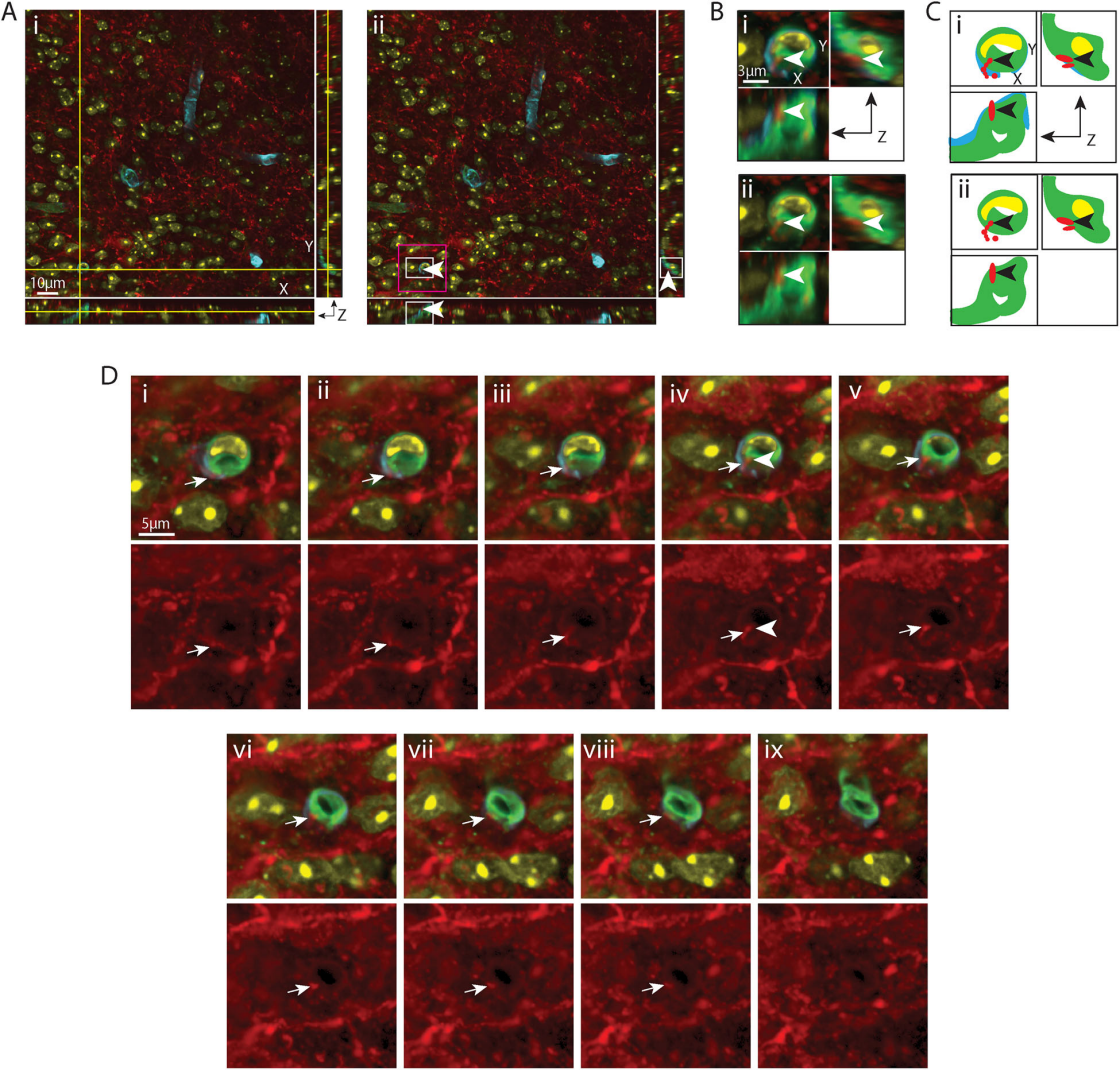
VIP fibers varicosity lying in the capillary endothelium. ***A***, A VIP varicosity in the endothelium is shown in all three orientations in an optical section taken from Figure 8*B*. ***i***, The crosshair denotes the location of the varicosity in the endothelium. ***ii***, Removal of the crosshair allows better visualization of the varicosity (arrowheads), which has a diameter of 0.65 µm. Higher magnification images of the white and magenta boxed areas are further examined in ***B*** and ***D***, respectively, in order to follow the path of the fiber. ***B***, Magnified view of the varicosity in all three orientations allows visualization of the varicosity in relation to the vascular endothelium. ***i***, VIP, collagen, lectin, and DAPI staining are merged to show that the varicosity is in the endothelium. ***ii***, Removal of collagen to show the varicosity is not localized in the basal lamina. Arrowheads point to the location of the varicosity as in ***A***. ***C***, Cartoon provides traces of key elements in ***B***. ***D***, Serial sections indicate the pathway (***i*–*iii***) of a VIP fiber (arrows). The varicosity (***iv***) terminates in the endothelium (***v*–*ix***). Top row: Merged images of VIP and BV show the path of the fiber. Bottom row: The VIP-only channel enhances visualization of the fiber. ***i*–*iii***, A VIP fiber appears and reaches the basal lamina of a capillary from the 7 o’clock direction. ***iv*–*vi***, The terminal of VIP fiber further enters the endothelium. ***vi*–*ix***, The varicosity gradually disappears. The arrowhead in ***iv*** serves as a location marker and points to the varicosity seen in ***A*** and ***B***. Color coding and abbreviations as in Figure 7. For ***A***, ***B***, and ***D***, *z* = 0.15 µm; ***D*** was taken from every fourth section across the *z* stack.

We first asked whether AVP, VIP, and GRP contacts with blood vessels are localized to distinct areas within the volume of the nucleus. The results indicate that processes of AVP, VIP, and GRP neurons contact capillary vessels wherever their fibers and varicosities are located, in both shell and core regions of the SCN. Varicosities of each of the peptidergic types make appositions with three distinct BV wall compartments, namely, the basal lamina matrix, the pericytes (identified by a DAPI-labeled nucleus), and the endothelium (Figs. 2–10). The figures present representative examples of AVP appositions (Figs. 2–4), followed by those of GRP (Figs. 5–7) and VIP (Figs. 8–10) in turn. For each of the vasoactive peptides, we present a set of three figures showing the following results: a varicosity within the basal lamina matrix, a varicosity contacting a presumed pericyte (based on its location within the basal lamina), and finally, a varicosity within the endothelium.

Figures 2–10 are similarly organized. Detailed descriptions of each result are provided in the figure legends. In each figure, Panel A shows a sagittal view of the SCN and indicates the location of the varicosity that is described in detail in the remaining panels (Fig. 9*A* applies to both Figs. 9, 10). Panel B is a higher magnification image of the boxed area in Panel A and shows the dense arrangement of fibers coursing over blood vessels in the region of interest. Subsequent images identify the contact site in increasingly higher resolution images of the varicosity of interest and blood vessel compartments in all three axes (Figs. 2–9*C*,*D*, 10*A*,*B*). To facilitate the visualization of the contact site, each layer of the highest resolution confocal image is traced to make a diagram (Figs. 2–9*E*, 10*C*) using Illustrator (Adobe). In the final images, the pathway of the fiber process that gives rise to the varicosity of interest is shown in a series of confocal images in *z* stacks. This is done by tracking the fiber as it courses toward the capillary vessel. In some cases (Figs. 2, 4, 5, 8), the fiber is en passant while in other cases it terminates at the point of contact with a BV compartment. It is noteworthy that in each case, fibrous varicosities access compartments within capillary vessels at points where there are gaps between astrocytic end feet. These occur at points where collagen staining is much reduced or absent (Figs. 2, 6, 7*D*).

## Discussion

Historically, it has been suggested that the BBB acts as a “physical barrier” because of the tight junctions between adjacent endothelial cells (Abbott et al., 2006). Also, brain capillaries are thought to be surrounded by, or closely associated with, the end feet of astrocytes which creates an additional barrier. In the current study, we raise the possibility that the varicosities of all three vasoactive peptides in the SCN access the basal membrane and endothelium indicating that there are gaps in the astrocytic coverage of capillary vesicles.

Astrocytic end feet are a component of the BBB. The experimental evidence in the literature on the extent of coverage and on the frequency of direct contacts between astrocytes and brain blood vessels ranges surprisingly widely, from 70 to 100%. In some estimates, astrocyte end feet outside the basement membrane are said to surround ∼85% of the surfaces of brain capillaries (Bai and Zhou, 2017; Al-Huthaifi et al., 2024). Electron microscopy (EM) in humans showed that the capillary coverage by glia is 85.15 ± 14.37% (Bertossi et al., 1997). Pardridge (2002) commented that >99% of the brain surface of the capillary basement membrane is surrounded by astrocyte end feet processes with occasional neuronal innervation of the capillary. In a study using 3D reconstruction of a small volume of the rat hippocampal CA1 region, based on EM data, Mathiisen et al. (2010) (Fig. 2*C*) reported that there are clefts between astrocytic end feet and suggested that 1% of the pericytes in microvessels are free of astrocyte coverage. The BBB is modulated by pericytes lying in the basal membrane abluminal to the endothelium, and signaling from pericytes regulates the number of tight junctions between endothelial cells (Armulik et al., 2010). The permeability of BBB is impacted by all its components and their interaction with neurons and immune cells (Zhao et al., 2022). Of course, coverage of BV by astrocytes may be regionally differentiated, and astrocytes themselves are very diverse (Haim and Rowitch, 2017). These authors noticed small spaces in the end foot sheath which were occupied by cellular processes. The activity of astrocyte end feet in regulating components of the BBB is also highly regional and dynamic (Segarra et al., 2021; Díaz-Castro et al., 2023; Pociu¯te˙et al., 2024). The current results and the broader literature support the possibility that while the majority of brain vasculature is covered by the astroglial end feet, this may not be the case for the entire vasculature surface. The present study raises the question of whether spaces between the end feet allow neuronal processes to access the vasculature wall.

The major findings of the present study are consistent with the accumulating evidence that vasoactive fibers innervate brain vasculature. In the SCN, we observed varicosity contacts over each region of the SCN (Fig. 1) of all three vasoactive peptides that were examined. The distribution of these contacts seemed to be related to the density and location of the varicosities. The contacts of AVP varicosities with the capillaries were found mostly in the shell along the rostrocaudal extension of the SCN and were rarely found in the core. The VIP and GRP varicosities were found throughout the entire extent of SCN. We conjecture that AVP has greater functional importance in the dense shell capillaries, while GRP and VIP have a more general effect on the entire SCN vasculature. While the relationship of AVP, VIP, and GRP varicosities to the vasculature of the SCN has never been examined, the present results are consistent with prior findings in the literature in other brain regions. For AVP, brain microvessel innervation by neurons has been reported in rats (Jójárt et al., 1984). Based on peroxidase-antiperoxidase immunocytochemistry and EM, they reported that AVP-positive elements establish direct contacts with the basal lamina of the endothelium or of a pericyte associated with the capillary bed. Furthermore, the results demonstrated that the AVP-containing neural processes that contact BVs are mainly dendrites, suggesting that the vasopressinergic neuronal elements can directly innervate brain microvessels. VIP has been reported in the discontinuities of astrocyte end feet and in close contact with the basal lamina of microvessels in the rat cortex (Paspalas and Papadopoulos, 1998). In addition, there is substantial VIP innervation of brain arteries in rats and mice (Lorén et al., 1979; Eckenstein and Baughman, 1984; Suzuki et al., 1988). While there are few studies of this peptide, GRP terminals reportedly innervate pial vessels in cats, guinea pigs, rats, and mice (Uddman et al., 1983). Additionally, several studies point to capillary innervation by other transmitters or modulators in extra-SCN brain regions. Innervation of capillaries by local neurons has been demonstrated in the cat hypothalamus (Rennels et al., 1983). Such contacts are not limited to vasoactive peptides; there is ultrastructural evidence of central monoaminergic innervation of BVs in the paraventricular nucleus of the hypothalamus (Larsson et al., 1976; Swanson et al., 1977). Finally, in the hepatic portal system, there is ultrastructural evidence of substance P and VIP innervation of BV, which are known to play a role in the modulation of rhythmic contractions (Barja and Mathison, 2008).

A caveat in this study is that fixation and preparation can have huge effects on tissue morphology. Collapse of perivascular spaces upon death of the animal and fixation of the tissue have been documented (Mestre et al., 2018). Projections to the basal lamina of the capillaries may reach perivascular spaces and could implicate glymphatic movement (Silver et al., 2025). This interesting possibility requires functional/dynamic studies in living animals. Additionally, the present results rest on confocal imaging methods, and further multimodal approaches will be essential for studying the structural organization and dynamic properties of neural–vascular communication systems. Greater resolution, in studies using nanoscale imaging techniques, EM, including scanning, transmission, and volume EM, will enable higher resolution visualization of the ultrastructure of fiber contacts with the neurovascular unit. Each technique, however, has distinct advantages and limitations. EM is limited by a relatively small field of view and restricted capacity for simultaneous identification of multiple proteins (Malatesta, 2021; Peddie et al., 2022). Super-resolution microscopy techniques beyond confocal imaging, which overcome the diffraction limit, also offer powerful tools for studying vasoactive fiber contacts with the vasculature. Although these methods enable visualization of multiple proteins and can be applied to study the activities of vasoactive fibers in vivo, they rely on indirect signals from fluorescent labels rather than direct structural visualization. In addition, the high cost of implementing super-resolution microscopy has hindered its widespread application (Schermelleh et al., 2019; Fuhrmann et al., 2022).

While it has not been studied directly, it is possible that the vasoactive peptides of the SCN act on the receptors in the vasculature. AVP receptors are widely distributed in the brain BV (Ostrowski et al., 1994). Upon brain injury, significantly increased AVP V_1a_ receptors localized on the vascular endothelium increase BBB permeability (Szmydynger-Chodobska et al., 2004). In Brattleboro rats, vasopressin binding sites show in the brain BVs in AVP-implanted animals but not in water-filled controls (van Zwieten et al., 1988). Furthermore, the staining was dose-dependent. The authors note that AVP binding on endothelial and pericyte cells points to a role in alteration of BBB permeability and involvement of pericytes in regulating blood flow in small vessels. While the evidence in Brattleboro rats is interesting but requires cautious interpretation, given their diabetic state and severely compromised physiology. VIP receptors have also been reported in brain BVs, and they are expressed exclusively in smooth muscle cells, not in the endothelium (Erdling et al., 2013). The same study also reported that only abluminal application of VIP could cause vascular relaxation. In an ischemia model with middle cerebral artery occlusion, intraventricularly administered VIP significantly reduced BBB permeability, shown by Evans Blue extravasation (Yang et al., 2022). To our knowledge, there is no report about the effect of GRP on brain vasculature. The present results point to the importance of exploring how SCN-derived neuropeptides regulate local hemodynamics and whether the hemodynamic changes impact the circadian regulation functions of the brain's clock.

There is limited information available on the localization of intra-SCN receptors for these vasoactive peptides. The mRNA of AVP receptors V1a and V1b is present in the SCN (Bedont et al., 2018). V1a shows strong expression in the central SCN and has a clear diurnal rhythm, peaking in the middle of the dark phase. It also displays differential expression along the rostrocaudal axis, with higher expression in the caudal SCN and lower in the rostral region when standardized to the entire SCN. In contrast, V1b is expressed at lower levels in the central SCN and is not rhythmic. There are two types of VIP receptors in the central nervous system, termed VPAC1 and VPAC2. Within the SCN, VPAC1 is not detected in SCN neurons but is extensively expressed in the vasculature (Stangerup and Hannibal, 2020; Fig. 1*D*). In contrast, VPAC2 is broadly distributed throughout the SCN, with higher expression in the dorsal region than in the ventral area (An et al., 2012). The same study also reported that approximately 94.4% of cultured SCN cells express VPAC2, primarily localized to dendrites and cell bodies. In another study, VPAC2 mRNA was identified in nearly all AVP neurons and 41% of VIP neurons (Kalamatianos et al., 2004). VPAC2 mRNA also exhibits diurnal rhythmicity, reaching a trough in the middle of the light phase (Kalló et al., 2004). The GRP receptor is likewise expressed in the SCN. Karatsoreos et al. (2006) demonstrated that GRP receptors are distributed across the rostrocaudal extent of the nucleus, with localization predominantly in the dorsal and medial SCN and lower expression in the ventrolateral region. They further reported that GRP receptor binding activity peaks at the onset of the dark phase, paralleling the oscillatory pattern of GRP receptor protein levels in the SCN. Finally, to the best of our knowledge, there are no data on whether there are receptors for AVP or GRP within the SCN vasculature.

The SCN–OVLT portal venules constitute a possible pathway for delivering peptides of SCN origin to the OVLT. Of the three vasoactive peptides studied here, AVP is the most likely to be involved in the SCN–OVLT portal communications. The portal vessels originate at the rostral shell of the SCN, a region rich in AVP. Single-cell RT-PCR revealed AVP V1a receptors in OVLT neurons (Gizowski et al., 2016), implying that OVLT is a target for this peptide. In addition, there are carrier-mediated systems transporting AVP from the brain to the blood (Banks et al., 1987). Although it has not been examined in the SCN, in other brain regions, peptides can enter the vasculature by efflux transport (Cox et al., 2023) and by transcytosis (Ayloo and Gu, 2019).

While the present study is strictly anatomical, it provides a solid foundation for future mechanistic work. The results suggest that varicosities of all three vasoactive peptides are found in the SCN vascular basal lamina and endothelium, pointing to the possibility that they may breach the BBB. This finding opens the opportunity for studying local vascular regulation within the nucleus. Given the portal connection between SCN and OVLT, these vasoactive peptides are positioned to modulate SCN hemodynamics and modulate diffusible signal efficacy. Possibly, neurosecretions relayed from the SCN to the OVLT and thence to other brain regions thereby provide for global signaling supporting daily rhythms (Silver et al., 2025).

## Synthesis

Reviewing Editor: Charlotte Cornil, Universite de Liege Decisions are customarily a result of the Reviewing Editor and the peer reviewers coming together and discussing their recommendations until a consensus is reached. When revisions are invited, a fact-based synthesis statement explaining their decision and outlining what is needed to prepare a revision will be listed below. The following reviewer(s) agreed to reveal their identity: NONE.

Your manuscript has now been reviewed by two experts in the field and myself. We all agree that the anatomical mapping of peptide-immunoreactive terminals in the mouse SCN and their proximity to capillary structures provides advances that are informative to move the field of circadian biology forward. The imaging is beautiful and convincing and the observations are supported by previous literature in other brain regions. However, there are some very large caveats and over-interpretations of findings that bring great concern. Notably, several statements go beyond what the data support, especially the claim that these terminals breach the blood-brain barrier or access the luminal blood. Moreover, the work presented is only qualitative (and actually not very detailed).

To advance the field, the manuscript should be reframed as an anatomical description with quantified analyses of terminal-capillary associations and clearly stated limitations due to the resolution of light microscopy. The authors are encouraged to (1) include quantitative metrics (e.g., distribution and proximity of terminals to capillaries across SCN subregions), (2) temper conclusions to reflect anatomical rather than functional inferences, and (3) outline concrete next steps (e.g., higher-resolution imaging, electron microscopy, or functional assays) that would test whether any vascular exchange or BBB breach actually occurs. Such revisions would make a meaningful contribution by providing a solid anatomical foundation for future mechanistic work.

Specific comments:

Advances to the field:

This manuscript does provide advances to the field of circadian biology. Specifically, it addressed how diffusible factors may extend beyond traditional SCN-extraSCN neuronal networks. The innervations of AVP, VIP, and GRP neurons to the basal lamina, pericytes, and endothelial cells of the capillaries within the SCN is fundamental anatomy that is necessary for the field to move forward. The results could have large implications for how the clock center of the brain communicates with the rest of the brain and periphery.

Over-interpretations:

- Although the neuron-to-capillary contacts exist, this does not mean they are "breaching the blood-brain barrier" as the title suggests. At best, they are "breaching the neurovascular unit".

- The discussion on how the BBB is opening from these neuronal inputs is wildly speculative and needs to be clearly addressed as such.

- Similarly, the evidence in Brattleboro rats is a bit over-stated as these animals are diabetic and have multiple things wrong with their physiology that could impact BBB permeability independent of these neuronal projections. Isn't it much more likely that these vasoreactive compounds are altering vascular pulsatility? Again, to answer this may be beyond the scope of this manuscript but this possibility should be at least mentioned in the discussion.

- The authors use traditional paraformaldehyde fixation and sucrose cryoprotection to prepare these brain slices. Fixation and preparation can have huge effects on astrocyte morphology, and can drive differences in astrocyte coverage estimates from 64 - 100% depending on technique. Therefore, the discussion on astrocyte coverage and how gaps in endfeet may allow for neuronal projections is hugely overstated speculation.

- At no point do the authors address that upon death of the animal and fixation of the tissue the perivascular space collapses. This is interesting because the projections to the basal lamina of the capillaries may actually be the perivascular space and could implicate glymphatic movement. This caveat and alternative hypothesis would hopefully be addressed in revisions, as fixation method and postmortem analysis are the biggest caveat of this manuscript.

Lack of quantitative data:

- In terms of the data presented, although the authors state that there was sufficient animal number there was no quantification of anything in this manuscript. We don't know how many capillaries were innervated by which peptide, quantification of which regions this happened in, if this was consistent across animals, or if there was any kind of trend based on sex of the animal (even though sex differences have been observed with several of these peptides including AVP). While the qualitative evidence is certainly intriguing, it is hard to understand if this may be a critical and meaningful input to control vasculature without a little more information. Based on the following information provided page 7 "Two independent investigators examined an EDF image of each square in the grid for potential points of contact between neurons and capillary vessels", it seems that the quantification has been done. It should thus be reported and presented in a way that allows to appreciate the degree of variation across individuals, sexes and sub-regions of SCN.

Proof of antibody validation

- Although all used antibodies are properly identified, information regarding their validation is lacking. Please provide reference to prior studies reporting the measures to insure antibody specificity or the steps you took to validate them if it had not been done before.

Minor comments:

- Line 42: what is meant by "nor in endocrine" is not clear.

- Line 48 : problem with citation formatting. To be checked throughout

## Author Response

Dec 29, 2025 Dear Dr Cornil, All authors thank you heartily for the positive review of this manuscript and for the constructive comments that have been provided. In this letter, we summarize all the updates and corrections. The summaries and reviewer comments are shown here in normal font, and our responses are presented in underlined italics. The changes in the main text are in red font.

Comments from the editor:

Your manuscript has now been reviewed by two experts in the field and myself. We all agree that the anatomical mapping of peptide-immunoreactive terminals in the mouse SCN and their proximity to capillary structures provides advances that are informative to move the field of circadian biology forward. The imaging is beautiful and convincing and the observations are supported by previous literature in other brain regions.

-Thank you for these laudatory comments.

However, there are some very large caveats and over-interpretations of findings that bring great concern. Notably, several statements go beyond what the data support, especially the claim that these terminals breach the blood-brain barrier or access the luminal blood.

-As suggested, we have revised the statement that the "terminals breach the blood-brain barrier or access the luminal blood" and indicate that the work simply raises the possibility that the blood-brain interface has been breached. This also addresses the reviewer's concern (see below) with our use of the descriptor "blood-brain barrier".

Moreover, the work presented is only qualitative (and actually not very detailed).

-The essence of the work involves anatomical studies, and as such, it is descriptive. The prior note indicates that the images are beautiful and convincing and that is inconsistent with the comment here that the work is not detailed. We have presented each image of each contact is presented in detail, along with explanatory cartoons. The quantitative aspects are now provided in a new section in methods.

To advance the field, the manuscript should be reframed as an anatomical description with quantified analyses of terminal-capillary associations and clearly stated limitations due to the resolution of light microscopy.

- Thank you for this suggestion. The quantified analyses are now incorporated into methods. The limitations of the methods we used have been incorporated into the text, as suggested.

The authors are encouraged to (1) include quantitative metrics (e.g., distribution and proximity of terminals to capillaries across SCN subregions), -As shown in the manuscript, we surveyed the entire SCN in seeking contacts (Figure 1). As we wrote in the discussion section of the paper, each of the three peptides examined has a different distribution of neurons and fibers within the SCN. To make this clearer, we have now added the information on numbers fo terminals that were examined in one section of the paper, so that the metrics that were performed are more accessible to the reader. (2) temper conclusions to reflect anatomical rather than functional inferences - As suggested, we have focused the conclusions on anatomical inferences and made it clear that the functional consequences of the anatomical organization are of interest but unknown. (3) and, outline concrete next steps (e.g., higher-resolution imaging, electron microscopy, or functional assays) that would test whether any vascular exchange or BBB breach actually occurs. Such revisions would make a meaningful contribution by providing a solid anatomical foundation for future mechanistic work.

-In keeping with this suggestion, the following text has been added to the manuscript.

While the present study is strictly anatomical, it provides a foundation for future work. The results of the current study rest on confocal imaging methods, and further studies will be essential for understanding the structural organization and dynamic properties of neural-vascular communication systems. Greater resolution, in studies using nanoscale imaging techniques, electron microscopy (EM), including scanning, transmission, and volume EM, will enable higher resolution visualization of the ultrastructure of fiber contacts with the neurovascular unit (NVU). Multimodal approaches are needed as each technique has distinct advantages and limitations. EM is limited by a relatively small field of view and restricted capacity for simultaneous identification of multiple proteins (Malatesta, 2021; Peddie et al., 2022). Super-resolution microscopy techniques beyond confocal imaging, which overcome the diffraction limit, also offer powerful tools for studying vasoactive fiber contacts with the NVU. Although these methods enable visualization of multiple proteins and can be applied to study the activities of vasoactive fibers in vivo, they rely on indirect signals from fluorescent labels rather than direct structural visualization. In addition, the high cost of implementing super-resolution microscopy has hindered its widespread application (Fuhrmann et al., 2022; Schermelleh et al., 2019).

Specific comments:

Advances to the field:

This manuscript does provide advances to the field of circadian biology. Specifically, it addressed how diffusible factors may extend beyond traditional SCN-extraSCN neuronal networks. The innervations of AVP, VIP, and GRP neurons to the basal lamina, pericytes, and endothelial cells of the capillaries within the SCN is fundamental anatomy that is necessary for the field to move forward. The results could have large implications for how the clock center of the brain communicates with the rest of the brain and periphery.

-Thank you for this concise summary.

Over-interpretations:

- Although neuron-to-capillary contacts exist, this does not mean they are "breaching the blood-brain barrier" as the title suggests. At best, they are "breaching the neurovascular unit".

We thank the reviewer for raising this concern. We have changed the term blood brain barrier to the more appropriate descriptor, the blood-brain interface, in keeping with the writings of Banks and others) this terminology better reflects the "culture change" in current thinking about BBB and is a better descriptor (https://doi.org/10.1038/nrd.2015.21. (Galea, Ian, and V. Hugh Perry. "The blood-brain interface: a culture change." Brain, Behavior, and Immunity 68 (2018): 11-16.) - The discussion on how the BBB is opening from these neuronal inputs is wildly speculative and needs to be clearly addressed as such.

-The point that this idea is strictly speculation has been clearly incorporated. The possibility of modulatory effects on the vascular system are noted.

- Similarly, the evidence in Brattleboro rats is a bit over-stated as these animals are diabetic and have multiple things wrong with their physiology that could impact BBB permeability independent of these neuronal projections. Isn't it much more likely that these vasoreactive compounds are altering vascular pulsatility? Again, to answer this may be beyond the scope of this manuscript but this possibility should be at least mentioned in the discussion.

-The caveat indicating that there are limitations to the interpretation of findings in the Brattleboro rat has been incorporated.

- The authors use traditional paraformaldehyde fixation and sucrose cryoprotection to prepare these brain slices. Fixation and preparation can have huge effects on astrocyte morphology, and can drive differences in astrocyte coverage estimates from 64 - 100% depending on technique. Therefore, the discussion on astrocyte coverage and how gaps in endfeet may allow for neuronal projections is hugely overstated speculation.

-The present study does not explore astrocyte coverage. The point made in the discussion section is simply that we have very poor understanding of the extent of astrocyte coverage. The writing has been clarified to make this point. Understanding the extent of astrocyte coverage of blood vessels is important in the context of the present study, as fibers cannot penetrate astrocytic processes.

- At no point do the authors address that upon death of the animal and fixation of the tissue the perivascular space collapses. This is interesting because the projections to the basal lamina of the capillaries may actually be the perivascular space and could implicate glymphatic movement. This caveat and alternative hypothesis would hopefully be addressed in revisions, as fixation method and postmortem analysis are the biggest caveat of this manuscript.

-We thank the reviewer for this excellent point and the relevant literature is now cited in the revised manuscript.

Lack of quantitative data:

- In terms of the data presented, although the authors state that there was sufficient animal number there was no quantification of anything in this manuscript. We don't know how many capillaries were innervated by which peptide, quantification of which regions this happened in, if this was consistent across animals, or if there was any kind of trend based on sex of the animal (even though sex differences have been observed with several of these peptides including AVP). While the qualitative evidence is certainly intriguing, it is hard to understand if this may be a critical and meaningful input to control vasculature without a little more information. Based on the following information provided page 7 "Two independent investigators examined an EDF image of each square in the grid for potential points of contact between neurons and capillary vessels", it seems that the quantification has been done. It should thus be reported and presented in a way that allows readers to appreciate the degree of variation across individuals, sexes and sub-regions of SCN.

-As noted by the reviewer, the quantification has been performed in the course of the studies. In the revised manuscript, the available quantitative information is now provided in a separate section in the methods section, so that it is more accessible to the reader.

Proof of antibody validation - Although all used antibodies are properly identified, information regarding their validation is lacking. Please provide reference to prior studies reporting the measures to insure antibody specificity or the steps you took to validate them if it had not been done before.

-This has been done in the revision.

Minor comments:

- Line 42: what is meant by "nor in endocrine" is not clear.

- Line 48: problem with citation formatting. To be checked throughout -This has been done in the revision.

Correction: The address for Dr. Yao has been corrected. The work was performed at the Columbia Psychology Department and NYSPI is her current address Yifan Yao1,2, Isabella Cannova1, Ruya Tzebay3, Saphira Samuels3, Emily Silverstein2, Rae Silver2,3,4,* Sincerely, Rae Silver on behalf of all authors E-mail: rae.silver@columbia.edu Department of Psychology, Columbia University
